# Author Correction: Discovering highly potent antimicrobial peptides with deep generative model HydrAMP

**DOI:** 10.1038/s41467-023-40879-6

**Published:** 2023-08-23

**Authors:** Paulina Szymczak, Marcin Możejko, Tomasz Grzegorzek, Radosław Jurczak, Marta Bauer, Damian Neubauer, Karol Sikora, Michał Michalski, Jacek Sroka, Piotr Setny, Wojciech Kamysz, Ewa Szczurek

**Affiliations:** 1 Faculty of Mathematics, Informatics and Mechanics, University of Warsaw, Stefana Banacha 2, 02-097 Warsaw, Poland; 2 NVIDIA, 2788 San Tomas Expressway, Santa Clara, CA 95051 USA; 3 Department of Inorganic Chemistry, Faculty of Pharmacy, Medical University of Gdańsk, Al. Gen. J. Hallera 107, 80-416 Gdańsk, Poland; 4 The Centre of New Technologies, University of Warsaw, Stefana Banacha 2c, 02-097 Warsaw, Poland

**Keywords:** Machine learning, Protein design, Computational models

Correction to: *Nature Communications* 10.1038/s41467-023-36994-z, published online 15 March 2023

In the original version of this article, Fig. 2 was inadvertently duplicated from Fig. 5. The correct version of Fig. 2 is:
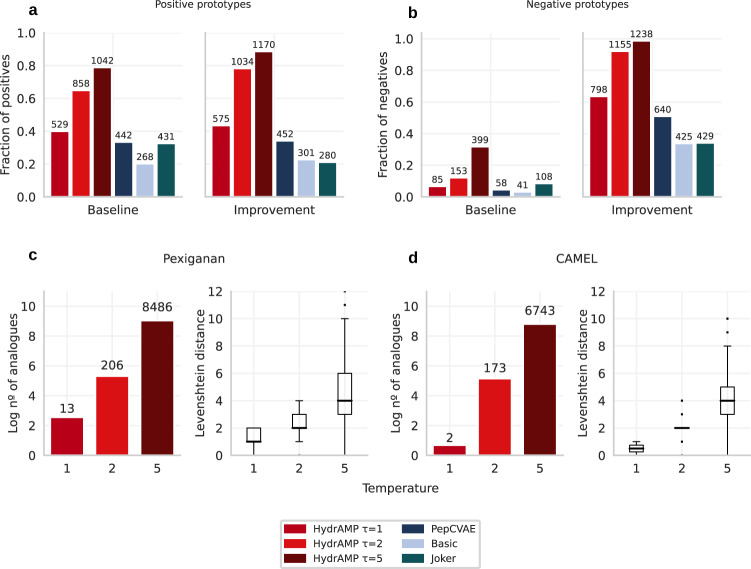


which replaces the previous incorrect version.

This has been corrected in both the PDF and HTML versions of the Article.

