# Supplementary material for: Discovering highly potent antimicrobial peptides with deep generative model HydrAMP
Source: Nat Commun. 2023 Mar 15;14:1453. doi: 10.1038/s41467-023-36994-z (PMC10017685; doi:10.1038/s41467-023-36994-z)
Supplement: Supplementary file 3 — Description of Additional Supplementary Files [file 41467_2023_36994_MOESM3_ESM.pdf]

Description of Additional Supplementary Files

Supplementary Data 1

Supplementary Data 1A: List of methods for the AMP generation, and the scope of their reproducibility considering the availability of: code, trained model, training data, and/or generated peptides.

Supplementary Data 1B: List of methods for the AMP classification, and the scope of their reproducibility considering the availability of: code, trained model, training data, and/or web service.

Supplementary Data 1C: List of methods for the toxicity prediction, and the scope of their reproducibility considering the availability of: code, trained model, training data, and/or web service.

Supplementary Data 2

Supplementary Data 2: List of peptides used as prototypes in the experimental phase of HydrAMP validation. The table contains information on: sequence, the antimicrobial activity of peptides against *E. coli, S. aureus, K. pneumoniae, P. aeruginosa*; toxicity against human erythrocytes, and the result of clinical trials (if applicable).

Supplementary Data 3

Supplementary Data 3: Peptides used for MD simulation calibration of S parameter. The table contains information on the peptide sequence, activity against E. coli and S. aureus, toxicity (HC50), as well as available structure (UniProt, PDB), and the results of full atomistic molecular dynamics simulations.

Supplementary Data 4

Supplementary Data 4A: Candidate analogues of GQ20 obtained through analogue generation selected for MD simulation.

Supplementary Data 4B: Candidate analogues of OP-145 obtained through analogue generation selected for MD simulation.

Supplementary Data 4C: Candidate analogues of Syphaxin obtained through analogue generation selected for MD simulation.

Supplementary Data 4D: Candidate analogues of Omiganan obtained through analogue generation selected for MD simulation.

Supplementary Data 5

Supplementary Data 5A: Experimentally validated analogues of GQ20 obtained through analogue generation combined with additional targeted preselection.

Supplementary Data 5B: Experimentally validated analogues of Syphaxin obtained through analogue generation combined with additional targeted preselection.

Supplementary Data 5C: Experimentally validated analogues of OP-145 obtained through analogue generation combined with additional targeted preselection.

Supplementary Data 5D: Experimentally validated analogues of Omiganan obtained through analogue generation combined with additional targeted preselection.

Supplementary Data 5E: Experimentally validated analogues of Pexiganan obtained through analogue generation without additional preselection and tested against additional strains.

Supplementary Data 5F: Experimentally validated analogues of Pexiganan and Temporin-A obtained through analogue generation without additional preselection.

Supplementary Data 6

Supplementary Data 6: Comparison of success rates of HydrAMP and other models (from Das et al. and Porto et al.) with success rate ratios compared to HydrAMP success rate.

Supplementary Data 7

Supplementary Data 7: Values of HC50, MIC and MBC in μM/ml for peptides generated by HydrAMP estimated as in https://pubmed.ncbi.nlm.nih.gov/29307075/.

Supplementary Data 8

Supplementary Data 8: The dedicated activity dataset used for AMP classifier evaluation. Second column indicates the origin of the collected sequences: dbaasp_ecoli - peptides from DBAASP database, active against E. coli ATCC 25922; uniprot - peptides filtered from UniProt database, assumed inactive against E. coli; inhouse - peptides from our in-house dataset of experimentally validated inactive peptides

Supplementary Data 9

Supplementary Data 9: The dedicated toxicity dataset used for toxicity classifier evaluation.
